# Insight into the Origin of Trapping in Polymer/Fullerene Blends with a Systematic Alteration of the Fullerene to Higher Adducts

**DOI:** 10.1021/acs.jpcc.1c10378

**Published:** 2022-01-31

**Authors:** Jose Marin-Beloqui, Guanran Zhang, Junjun Guo, Jordan Shaikh, Thibaut Wohrer, Seyed Mehrdad Hosseini, Bowen Sun, James Shipp, Alexander J. Auty, Dimitri Chekulaev, Jun Ye, Yi-Chun Chin, Michael B. Sullivan, Attila J. Mozer, Ji-Seon Kim, Safa Shoaee, Tracey M. Clarke

**Affiliations:** † Department of Chemistry, University College London, Christopher Ingold Building, London WC1H 0AJ, United Kingdom; ‡ ARC Centre of Excellence for Electromaterials Science, Intelligent Polymer Research Institute, University of Wollongong, North Wollongong, NSW 2500, Australia; § Institute of High Performance Computing A*STAR, Singapore 138632, Singapore; ∥ Optoelectronics of Disordered Semiconductors, Institute of Physics and Astronomy, University of Potsdam, Karl-Liebknecht-Strasse 24-25, Potsdam-Golm 14476, Germany; ⊥ Department of Chemistry, The University of Sheffield, Sheffield S3 7HF, United Kingdom; # Department of Physics and Centre for Processable Electronics, Imperial College London, London SW7 2AZ, United Kingdom

## Abstract

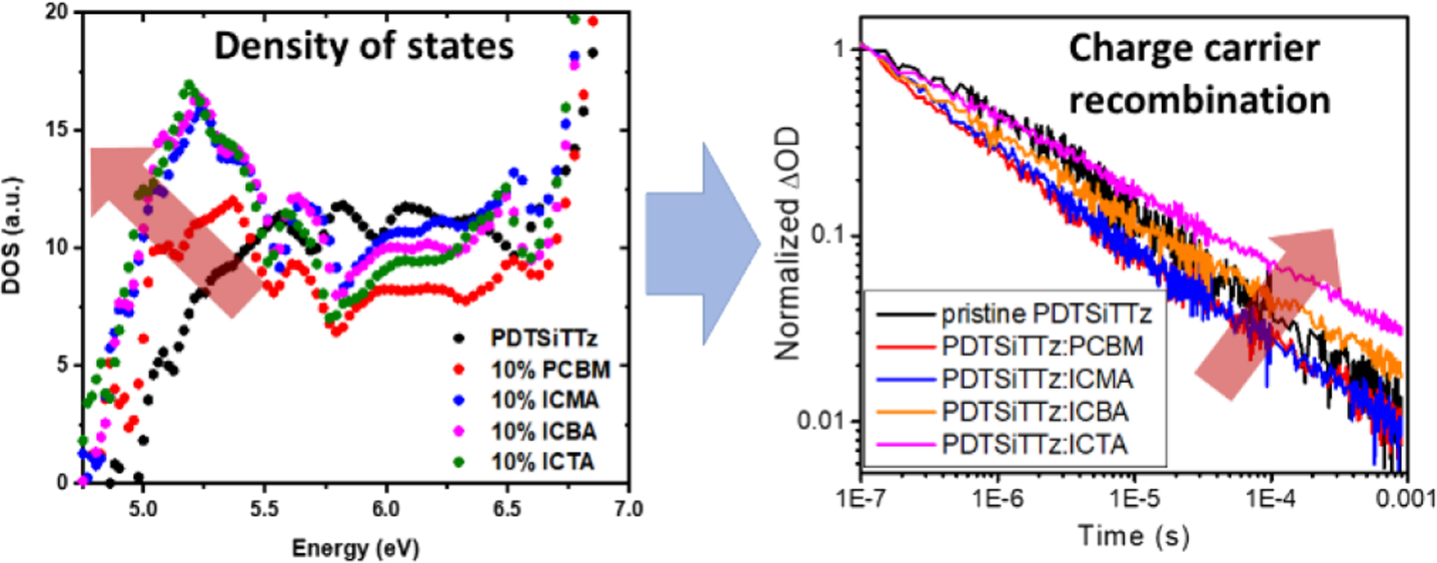

The bimolecular recombination characteristics of conjugated polymer poly[(4,4′-bis(2-ethylhexyl)dithieno[3,2-*b*:2′,3′-*d*]silole)-2,6-diyl-alt-(2,5-bis 3-tetradecylthiophen-2-yl thiazolo 5,4-*d* thiazole)-2,5diyl] (PDTSiTTz) blended with the fullerene series PC60BM, ICMA, ICBA, and ICTA have been investigated using microsecond and femtosecond transient absorption spectroscopy, in conjunction with electroluminescence measurements and ambient photoemission spectroscopy. The non-Langevin polymer PDTSiTTz allows an inspection of intrinsic bimolecular recombination rates uninhibited by diffusion, while the low oscillator strengths of fullerenes allow polymer features to dominate, and we compare our results to those of the well-known polymer Si-PCPDTBT. Using μs-TAS, we have shown that the trap-limited decay dynamics of the PDTSiTTz polaron becomes progressively slower across the fullerene series, while those of Si-PCPDTBT are invariant. Electroluminescence measurements showed an unusual double peak in pristine PDTSiTTz, attributed to a low energy intragap charge transfer state, likely interchain in nature. Furthermore, while the pristine PDTSiTTz showed a broad, low-intensity density of states, the ICBA and ICTA blends presented a virtually identical DOS to Si-PCPDTBT and its blends. This has been attributed to a shift from a delocalized, interchain highest occupied molecular orbital (HOMO) in the pristine material to a dithienosilole-centered HOMO in the blends, likely a result of the bulky fullerenes increasing interchain separation. This HOMO localization had a side effect of progressively shifting the polymer HOMO to shallower energies, which was correlated with the observed decrease in bimolecular recombination rate and increased “trap” depth. However, since the density of tail states remained the same, this suggests that the traditional viewpoint of “trapping” being dominated by tail states may not encompass the full picture and that the breadth of the DOS may also have a strong influence on bimolecular recombination.

## Introduction

Bimolecular recombination is one of the most significant loss mechanisms in organic photovoltaics (OPV). One of the primary reasons that organic photovoltaic systems lag behind in terms of power conversion efficiency compared to perovskite and inorganic solar cells is the low dielectric constant inherent in organic materials. The low dielectric constant causes a strong Coulombic attraction between opposing charges, affecting both charge photogeneration and bimolecular recombination.

It is therefore important to explore avenues to reduce detrimental recombination pathways in organic photovoltaics. To accomplish this, detailed knowledge of bimolecular recombination and how it operates in such systems is paramount. One issue with examining bimolecular recombination accurately is that there are numerous factors that can strongly influence recombination simultaneously. It is therefore difficult to isolate and alter only one of these parameters without affecting the others. One critical factor is diffusion. Most polymer/acceptor blends follow the Langevin model, in which recombination is controlled not by the intrinsic electron transfer rate between the recombining electron and hole but by the probability of the two opposite charges encountering one another: diffusion. However, there do exist in the literature a few examples of blend systems that display non-Langevin characteristics, in which recombination is not controlled by diffusion. For example, the polymer poly[(4,4′-bis(2-ethylhexyl)dithieno[3,2-*b*:2′,3′-*d*]silole)-2,6-diyl-alt-(2,5-bis 3-tetradecylthiophen-2-yl thiazolo 5,4-*d* thiazole)-2,5diyl] (PDTSiTTz, also known as KP115^1−3^) is known to be one of the few reported strongly non-Langevin polymer/fullerene systems in the literature, with very long-lived charge carriers and power conversion efficiencies of almost 5%. While this is a low efficiency relative to the most recent advances in OPV, PDTSiTTz remains of interest for bimolecular recombination studies since it is not limited by diffusion.

A second issue critical to accurate measurement of recombination is blend morphology, particularly at the blend ratios used for optimal solar cell performance. For PDTSiTTz:PC60BM, the optimal ratio is 1:2. At such high loadings of fullerene, a complex morphology is created, where crystalline domains of pure polymer and pure fullerene exist alongside more closely inter-mixed amorphous domains. To minimize the effects of such complex nanostructuring on bimolecular recombination, a small fullerene loading is essential. The fullerene ratio must be chosen such that a balance is provided between allowing exciton dissociation to take place but without perturbing the natural morphology of the pristine polymer.^4^

An important feature inherent to conjugated polymers is the presence of significant energetic and morphological disorder. The conjugated network is not likely to be maintained over the entire backbone, instead extending over a small chain segment. This inability to maintain long-range order is the result of kinks, chemical defects, and torsion around single covalent bonds found on the polymer backbone. This therefore leads to a range of conjugation lengths and consequently a distribution of the highest occupied molecular orbital (HOMO) and lowest unoccupied molecular orbital (LUMO) energies (energetic disorder), which plays an important role in dictating the transport properties of the material. This energetic disorder can influence bimolecular recombination by allowing trapping of charge carriers. The conventional interpretation is that the broad density of states present in polymer/fullerene systems creates energetically deep “tail” states that essentially trap charges.^5,6^ These charges then need to be thermally activated out of these trap states to recombine, leading to non-second-order recombination kinetics and longer-than-expected charge carrier lifetimes. This can also have significant effects on charge carrier extraction in photovoltaic devices as not all charge carriers may be successfully extracted to contribute to the photocurrent.

**Figure 1:**
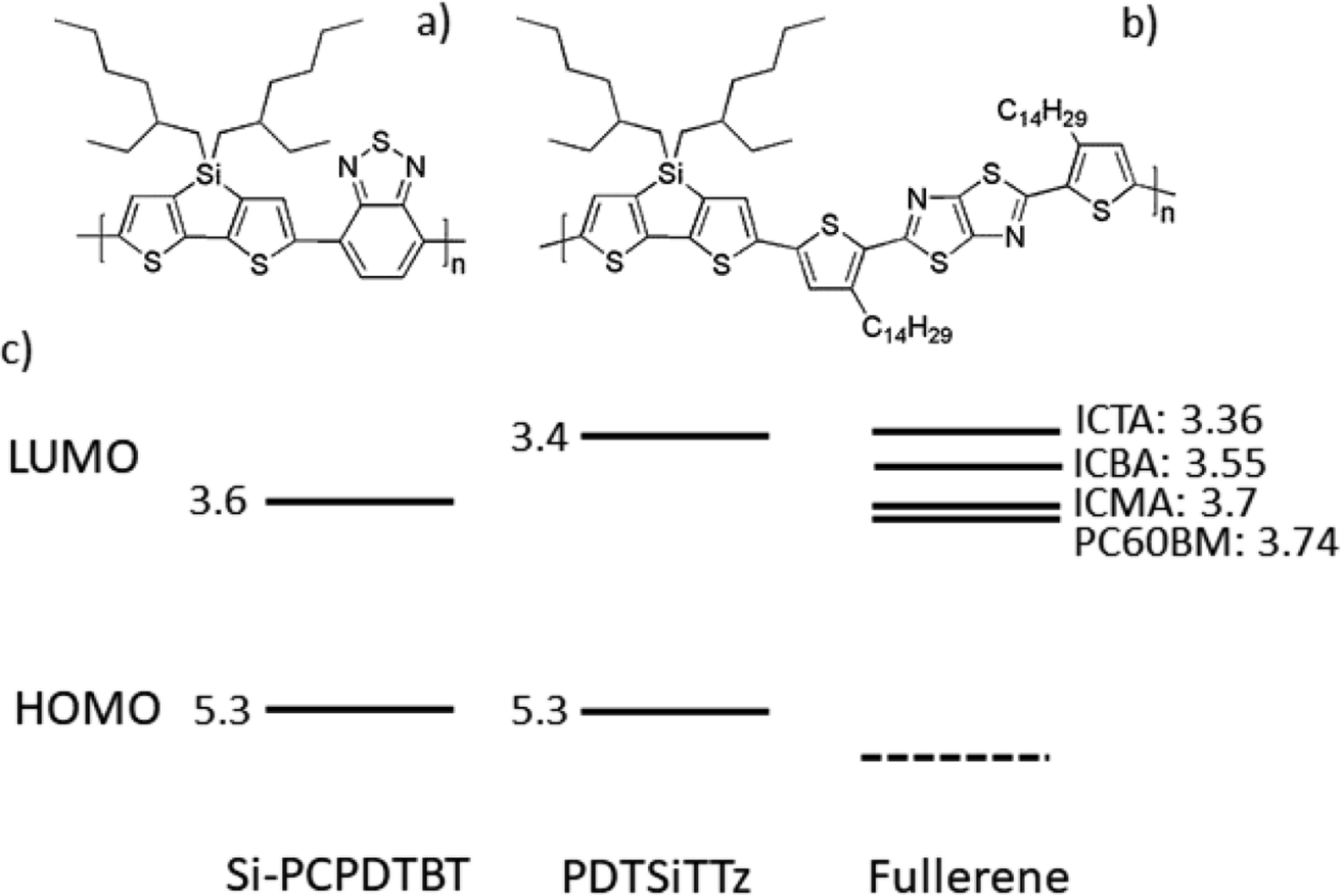
Structures of (a) Si-PCPDTBT and (b) PDTSiTTz and (c) the energy level diagrams of both polymers with the fullerenes used in this work (scale in eV). The HOMO of PDTSiTTz was established from cyclic voltammetry performed on thin film pristine samples (Supporting Information, Figure S1), and the LUMO was established by adding the optical band gap (determined through the intersection of absorption and fluorescence spectra normalized to the 0–0 transition). The HOMO of Si-PCPDTBT (CV on thin film) is taken from ref (15). The fullerene LUMO values are taken from ref (7).

In this paper, the effect of altering the acceptor on the trapping and bimolecular recombination behavior of polymer/fullerene blend films is assessed using transient absorption spectroscopy (TAS). TAS directly monitors the optical absorption of photogenerated transient species, providing information on the identity, yield, and recombination of these transient species. One strategy to explore bimolecular recombination is to alter the acceptor systematically. For example, the fullerene series PC60BM, ICMA, ICBA, and ICTA show a progression of the LUMO level toward the vacuum level (with values of −3.74, −3.7, −3.55, and −3.36 eV respectively,^7^ although it should be noted that these values vary slightly throughout the literature). Despite the current increase in popularity of non-fullerene acceptors, fullerenes remain an excellent choice for fundamental bimolecular recombination studies due to the wealth of literature available with regard to their spectroscopic characteristics and the weak oscillator strengths of their transient species,^8−11^ allowing the polymer transient species to dominate the spectra. In this work, the non-Langevin polymer PDTSiTTz (Figure 1) is compared with the well-known polymer Si-PCPDTBT, with which PDTSiTTz shares the donor unit and comparable crystallinity. To substantially reduce morphological effects for this spectroscopic study, a fullerene concentration of 10% (by weight) was used, and the identity of the fullerene acceptor was altered systematically from PC60BM to ICTA. The PDTSiTTz/ICTA combination is of particular interest due to a negligible LUMO level offset. Some systems with these marginal energy offsets—both fullerene and non-fullerene—are capable of unusually high charge photogeneration yields due to a hybridization between the local singlet exciton and charge transfer states,^12−14^ but much less is known regarding the effects of marginal energy offsets on bimolecular recombination.

Using μs-TAS, we show that the trap-limited decay dynamics of the PDTSiTTz polaron becomes progressively slower across the fullerene series, suggesting an increased “trap” depth, while those of Si-PCPDTBT are invariant. We explain these results using a combination of electroluminescence (EL) measurements and ambient photoemission spectroscopy (APS), from which the density of states (DOS) of the occupied states can be extracted. The EL measurements for pristine PDTSiTTz indicate an additional low energy intragap charge transfer state (likely interchain in nature) and a broad, low-intensity DOS. Unusually, the PDTSiTTz blends with ICBA and ICTA present a virtually identical DOS to Si-PCPDTBT, attributed to a shift from the delocalized, interchain HOMO in the pristine PDTSiTTz to a dithienosilole-centered HOMO in the blends. Although this HOMO localization was accompanied by a progressive shifting of the polymer HOMO to shallower energies, the density of tail states remained the same. As such, the strong correlation between the shifting of the HOMO band edge and the observed increased trap depth along the fullerene series suggests that the breadth of the main DOS peak, in addition to the tail states, may have a strong effect on trapping.

## Methods

### Spectroscopy Sample Preparation

All fullerenes were purchased from Solenne (>99% purity). PDTSiTTz was sourced from Organtec and the Si-PCPDTBT from Ossila. Solutions were prepared via dissolving the materials in spectroscopic grade dichlorobenzene (Alfa Aesar) and stirring overnight at 120 °C in a glovebox, with a N_2_ atmosphere. Thin films were prepared via spin-coating from solution. Glass substrates were cleaned via separately sonicating in solutions of deionized water, acetone, and isopropanol for 15 min each. Unless explicitly stated, all measurements were carried out under an inert atmosphere using either a continuous nitrogen flow or an evacuated Young’s tap cuvette.

### Steady State Absorption and PL

Absorbance spectra were recorded with a Perkin Elmer LAMBDA 365 UV–vis spectrophotometer. Fluorescence spectra were recorded with a Horiba FluoroMax-4 spectrofluorometer and corrected for instrument response at the exciting wavelength. Steady state spectra were recorded at room temperature.

### Ultrafast TAS

Transient absorption spectroscopy was performed at the Lord Porter Laser Laboratory, University of Sheffield. A Ti:sapphire regenerative amplifier (Spitfire ACE PA-40, Spectra-Physics) provided 800 nm pulses (40 fs FWHM, 10 kHz, 1.2 mJ). Pulses for excitation (520 and 665 nm) were generated from the fundamental 800 nm with a commercially available optical parametric amplifier (TOPAS, Light Conversion). White light super-continuum probe pulses in the range of 430–700 or 800–1500 nm regions were generated in situ using 2% of the Ti:sapphire amplifier output, focused on a CaF_2_ or YAG crystal, respectively. Detection was achieved using a commercial transient absorption spectrometer (Helios, Ultrafast Systems) using a CMOS sensor for the UV–vis or an InGaAs detector for NIR spectral range. The relative polarization of the pump and probe pulses was set to a magic angle of 54.7° for anisotropy-free measurements.

### Microsecond TAS

The encapsulated devices were excited in transmission mode by a laser pulse (6 ns, 532 nm, repetition rate of 10 Hz) from a Nd:YAG laser (Spectra-Physics, INDI-40-10) with a pump wavelength of 532 nm and a repetition frequency of 10 Hz. The Xe probe lamp (Edinburgh Instruments, Xe900) with a stabilized power supply was adjustable using a monochromator. The probe light passing through the device was detected with a silicon (Femto, HCA-S-200 M-SI) or an InGaAs photodiode (Femto, HCA-S-200 M-IN). The signal from the photodiode was amplified (Femto, DHPVA-200) and collected with a digital oscilloscope (Tektronics, DPO4054), which was synchronized with a trigger signal of the pump laser pulse from a photodiode (Newport, 818-BB-40). To reduce stray light, scattered light, and sample emission, appropriate optical cutoff and bandpass filters were placed before and after the sample.

### APS Measurements

Characterization of molecular energetics as well as density of states were carried by an APS04 system from KP Technology. Spin-coated samples of both neat and fullerene-blended Si-PCPDTBT and PDTSiTTz were prepared on top of ITO substrates. Samples were grounded with ITO throughout the measurements, providing a non-biased electrical background. Then, monochromatic UV light from a deuterium lamp was scanned from 4.5 to 7 eV on top of the thin film. The photoexcited electrons and radicals were collected by the positively biased tip. The cube roots of the APS signals were linear-fitted to the most linear region to get the HOMO level, and the DOS spectra were calculated by the energy derivative of the cube root signal.

### Device Preparation and Characterization

Patterned indium tin oxide (ITO) substrates were pre-cleaned in an ultrasonic bath with Hellmanex, deionized water, acetone, and isopropyl alcohol for 20 min for each of them and then dried by nitrogen. The cleaned substrates were treated by oxygen plasma at room temperature for 4 min at 200 W. After that, they were spin coated with nanoparticle ZnO (at 5000 rpm for 30 s) and then annealed at 120 °C for 20 min in air. Then the substrates were treated by UV light illumination for 10 min. The pristine PDTSiTTz and Si-PCPDTBT, and their 9:1 blends with PC60BM, ICMA, ICBA, and ICTA were dissolved in 1.2-dichlorobenzene with total concentration of 15 mg/mL and stirred overnight. After dissolving, the solutions were cooled down to room temperature. The active blend and pristine solutions were then spin coated (at 2000 rpm for 60 s) on top of the ZnO layer. The substrates were transferred into a vacuum evaporator connected to the glovebox, and 30 nm MoO_3_ with rate of 0.04 Å/s and 100 nm silver with rate of 0.6 Å/s were deposited sequentially through a shadow mask under ≈1 × 10^−7^ mbar, with an active area of the cells of A = 0.06 cm^2^. To measure the photovoltaic external quantum efficiency (EQE_PV_), the devices were illuminated by a monochromator (LOT-Oriel) with a 200 W halogen lamp. The output current of the devices was measured by a lock in amplifier (EG&G Princton Applied Research Model 5302). In front of the light source, an optical chopper was mounted as a reference frequency for the lock in amplifier. The light source was calibrated via a silicon photodiode (calibrated by Newport UV-818) and a germanium photodiode (calibrated by Newport 818-IR) for the visible and near infrared part of the light spectrum, respectively. To measure the external quantum efficiency of electroluminescence (EQE_EL_), charges were injected into the devices by a voltage-current source (Keithley 2400). The emission spectrum was detected under steady state conditions by an Andor SR393i-B spectrometer equipped with a silicon (Si) (DU420ABR-DD) and an indium-gallium arsenide (InGaAs) (DU491A-1.7) detector.

## Results

### Steady State Absorption and Photoluminescence Spectroscopy

**Figure 2:**
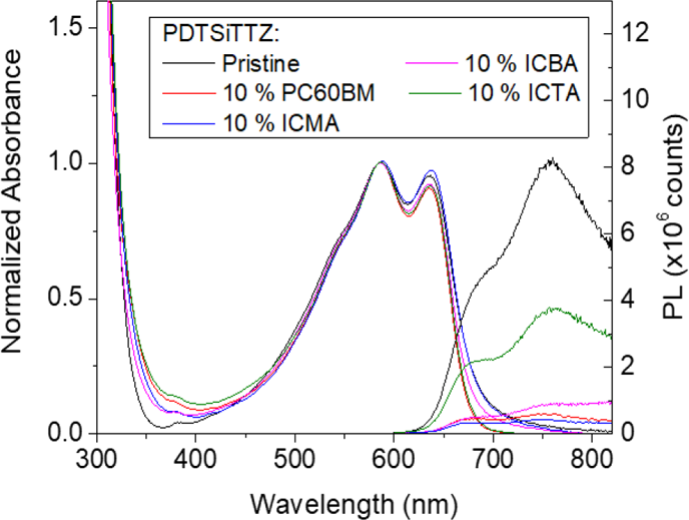
Steady state absorption and photoluminescence spectra for PDTSiTTz and its blends (9:1) with each fullerene studied. The photoluminescence spectra have been normalized to the absorbance at an excitation wavelength of 532 nm.

The steady state absorption spectra of pristine PDTSiTTz and its 9:1 blends with the varying fullerenes are shown in Figure 2. The polymer absorbance from 450 to 700 nm alters only slightly with the addition of fullerene, indicating that very little morphology change occurs. Indeed, it should be noted that these absorbance changes are substantially smaller than that observed in a PDTSiTTz:PC60BM 1:2 blend film, where the fullerene peak at 330 nm is also evident (Figure S2). Similarly, Si-PCPDTBT shows little change in its steady state absorption spectra with addition of fullerene (Figure S3a).

Photoluminescence (PL) spectroscopy was performed to ascertain the extent of exciton quenching in the 10% blend films. PDTSiTTz:PC60BM, PDTSiTTz:ICMA, and PDTSiTTz:ICBA all show quenching of the polymer emission by approximately 90%, indicating that the polymer exciton is efficiently quenched by the fullerene to create the polymer/fullerene charge transfer state (Figure 2). However, PDTSiTTz:ICTA shows less efficient PL quenching, with only 50% of the polymer emission quenched. Energetics is likely to play a significant role in this observation. The driving force for charge separation of PDTSiTTz:ICTA is almost zero (as estimated by the LUMO-LUMO offset), and thus, charge transfer from the polymer to the fullerene is energetically less favorable than for the other blends. In support of this argument, the PL quenching is still incomplete (65%) when the weight ratio of PDTSiTTz:ICTA blends is increased to 1:2, despite the abundance of fullerene in the blend film (Figure S4).

### Microsecond Transient Absorption Spectroscopy

**Figure 3:**
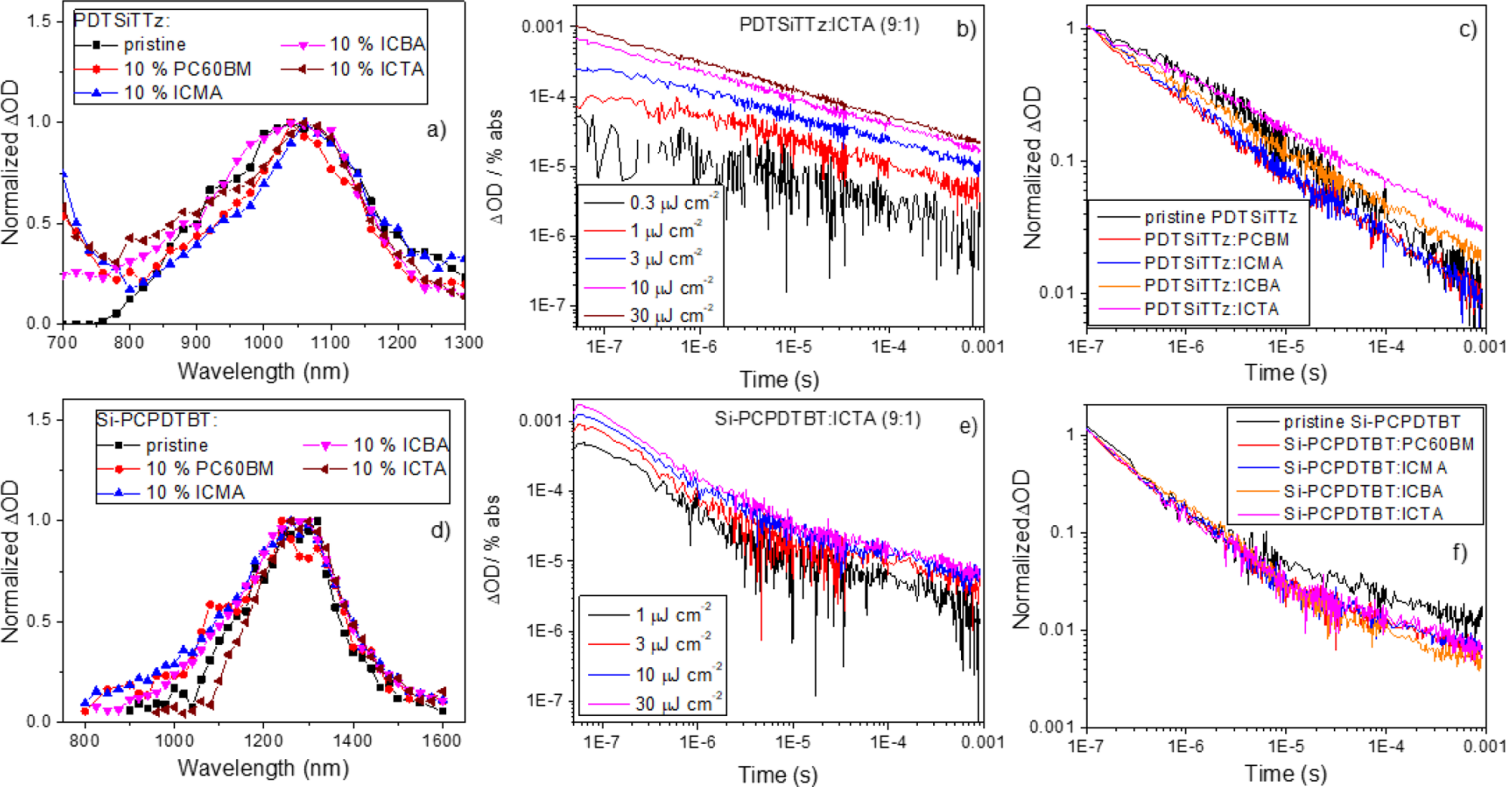
Microsecond transient absorption data for (a–c) PDTSiTTz and (d–f) Si-PCPDTBT. Pump excitation wavelengths of 532 and 600 nm were used for the PDTSiTTz and Si-PCPDTBT samples, respectively. The normalized transient absorption spectra at 1 μs are shown for (a) PDTSiTTz and (d) Si-PCPDTBT pristine polymers and their fullerene blends with 10% by weight. All spectra were measured using an excitation density of 10 μJ cm^–2^. The excitation density-dependent decay dynamics are shown for (b) PDTSiTTz:ICTA (9:1) and (e) Si-PCPDTBT:ICTA (9:1) films. Normalized decay dynamics are shown for (c) PDTSiTTz and (f) Si-PCPDTBT and their fullerene blend (9:1) films. Probe wavelengths of 1100 and 1300 nm (both 30 μJ cm^–2^) were used for the PDTSiTTz and Si-PCPDTBT kinetics, respectively. Bandpass filters were used in both cases.

The transient absorption spectra for PDTSiTTz and its 9:1 blends with the various fullerenes were measured; the normalized spectra at 1 μs are shown in Figure 3a and the kinetics in Figure 3b,c. Pristine PDTSiTTz is characterized by a single transient absorption band centered at 1050 nm. The power law decay dynamics of this band (Figure 3c) are consistent with that of bimolecular recombination of dissociated charge carriers in the presence of an exponential distribution of localized (trapped) states. The presence of polymer triplets can also be discounted on the basis of these power law kinetics (triplet decay should be mono-exponential); this was confirmed from the lack of oxygen sensitivity (Figure S5). The 1050 nm band is therefore assigned to the PDTSiTTz polaron. Since such a long-lived polaron in a pristine polymer is rather unusual, we investigated this further to check where the PDTSiTTz triplet is located. TAS of the PDTSiTTz solution showed the triplet to be present at 950 nm, and this was confirmed by assessing a PDTSiTTz:polystyrene (PS) matrix, where PS is an inert polymer (Figure S6). A PS matrix is used to simulate the polymer in a blend environment and also reduce PDTSiTTz aggregation and thus lengthen the transient species’ lifetimes. The PDTSiTTz:PS blend film produced evidence of both polarons and triplets, with the triplet still located at 950 nm (despite the more condensed phase) and the polaron at 1050 nm. Spectral evolution of the PDTSiTTz:PS blend clearly demonstrated that these two features have different kinetics, and thus we can be confident that the 1050 nm band in the pristine PDTSiTTz film is indeed the polymer polaron.

Addition of any of the fullerenes to create the PDTSiTTz:fullerene 9:1 blend films results in the 1050 nm transient absorption band and is therefore also assigned to the PDTSiTTz polaron, as reported previously.^16^ Unlike pristine PDTSiTTz, however, there is also a tail of absorption below 750 nm in all PDTSiTTz blends. Following oxygen-independent power law kinetics, this <750 nm band is also ascribed to a PDTSiTTz polaron. Since the 1050 nm band is also present for the pristine polymer, it is likely to involve bimolecular recombination occurring in predominantly pure (semi-crystalline) polymer regions. Conversely, the feature below 750 nm is influenced not by the fullerene identity but by its weighting. Both the relative amplitude and decay rate of the PDTSiTTz blends’ <750 nm band increase at a larger fullerene concentration of 1:2 (Figure S7).^16^ This <750 nm feature is therefore consistent with charge recombination occurring in a mixed phase including both polymer and fullerene domains. Recombination in this case would become faster as the availability of recombination sites (polymer/fullerene interfaces) increases, as observed. No clear evidence of PDTSiTTz triplets is observed in the fullerene blends, but a weak shoulder around 940 nm for the ICMA and ICTA blends may have a contribution from the PDTSiTTz triplet. A full discussion of these assignments is made in the Supporting Information.

The TA spectra for Si-PCPDTBT and its 9:1 blends with the various fullerenes are shown in Figure 3d and the kinetics in Figure 3e,f. The transient spectrum of pristine Si-PCPDTBT is characterized by a single peak centered at 1280 nm. Each blend transient spectrum shows the same, unshifted peak, which can be attributed to the Si-PCPDTBT polaron. Si-PCPDTBT:ICTA shows a much narrower band; however, this is most likely due to unquenched emission (which occurs at 900 nm) dampening the TA signal. Note, however, that the Si-PCPDTBT triplet absorbs in a very similar position to the polaron^17^ and that, while the kinetics are much more consistent with a polaron assignment, a contribution from the Si-PCPDTBT triplet state cannot be ruled out.

The excitation density dependence of the PDTSiTTz decay dynamics, probed at 1100 nm, are shown in the Supporting Information (Figure S8) for each 9:1 blend film, in addition to the pristine polymer. These are exemplified by PDTSiTTz:ICTA (9:1) in Figure 3b. One of the most notable differences as the fullerene is changed is the saturation of the slow phase. PDTSiTTz:PC60BM shows a much more rapid saturation of the slow phase than PDTSiTTz:ICTA (Figure S8). For PDTSiTTz:ICTA, the amplitude of the power law slow phase continues to increase markedly with excitation density even in the presence of the fast phase. This has previously been attributed to a significant fraction of shallowly trapped polarons under low excitation densities, even under conditions when the deep states are not fully occupied, therefore implying an incomplete thermalization of polarons on the timescale of bimolecular recombination.^18^

To assess the effect of altering the fullerene on the PDTSiTTz polaron decay kinetics, as probed at 1100 nm, they are normalized to 1 at 100 ns (Figure 3c). It is clear that the kinetics become progressively slower through the series from PDTSiTTz:PCBM to PDTSiTTz:ICTA. All have slower dynamics than the pristine polymer, for which the gradient of the power law decay, α, is 0.56. PDTSiTTz:PC60BM has an α value of 0.5, while PDTSiTTz:ICTA has α = 0.39.

The kinetics of the pristine Si-PCPDTBT and its blends are quite different compared to those observed for PDTSiTTz. A comparison of the normalized kinetics in Figure 3f shows that all of the Si-PCPDTBT:fullerene blends have virtually identical kinetics, with a slow phase α of 0.34. Furthermore, there is a marked difference in the saturation behavior of the slow phase between Si-PCPDTBT and PDTSiTTz (Figure 3e and Figure 3b, respectively). The slow phase of the Si-PCPDTBT saturates very rapidly with increasing excitation density, suggesting a complete thermalization of the charges, as exemplified by Si-PCPDTBT:ICTA in Figure 3e.

### Picosecond TAS

**Figure 4:**
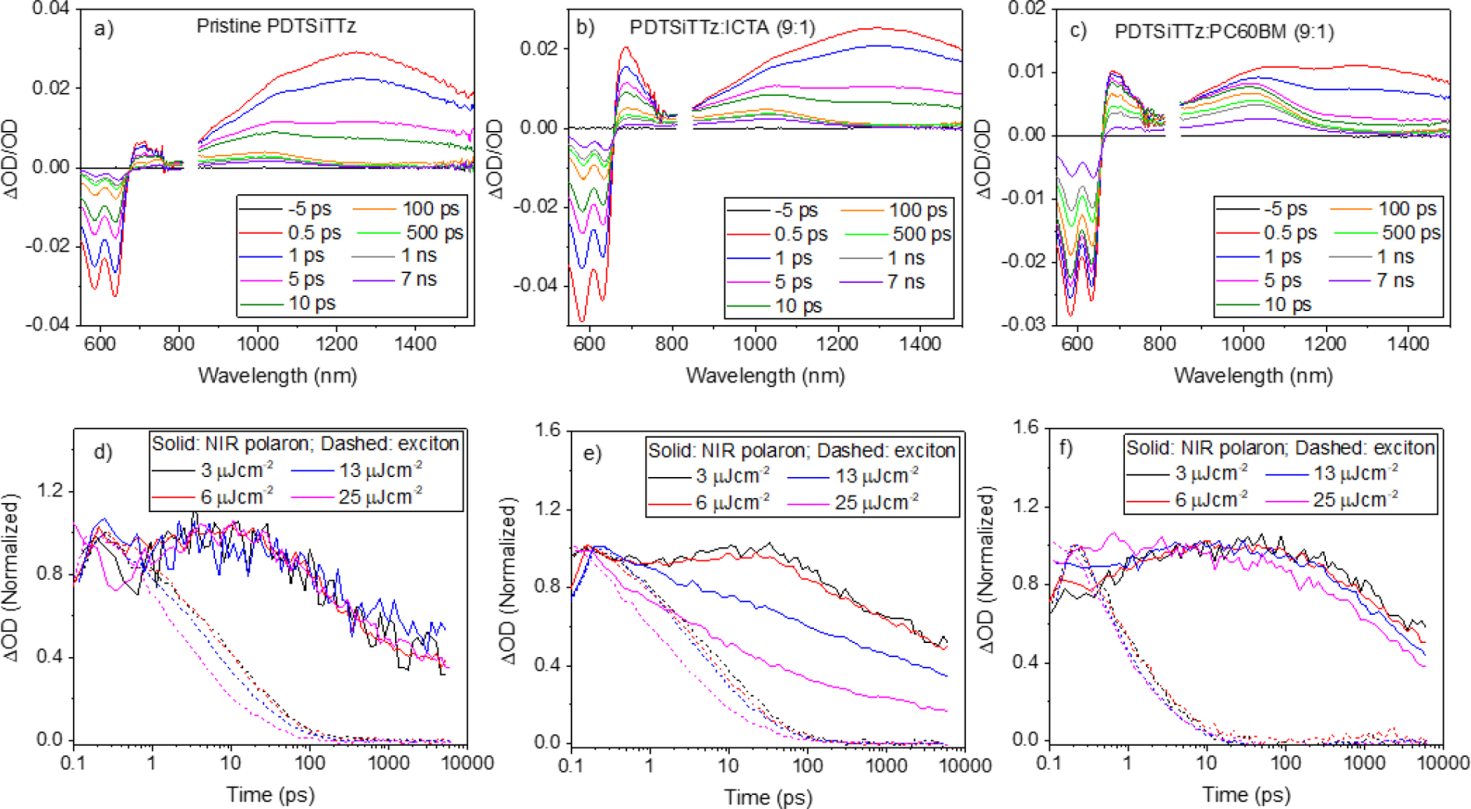
Femtosecond TA spectra and excitation density-dependent kinetics for (a, d) pristine PDTSiTTz, (b, e) PDTSiTTz:ICTA, and (c, f) PDTSiTTz:PC60BM films. The excitation wavelength was 532 nm in all cases, and an excitation density of 25 μJ cm^–2^ was used to obtain the TA spectra. The kinetics are derived from global analyses, where the exciton component absorbs maximally at 1270 nm and the polymer polaron at 1020 nm.

Given the intriguing microsecond TA behavior for PDTSiTTz and its fullerene blends (fullerene-dependent thermalization and kinetics and high charge carrier density for pristine and zero-offset systems), picosecond TAS was employed, and the results are shown in Figure 4. The pristine PDTSiTTz shows a strong singlet exciton (S_1_) peak at 1270 nm that decays to leave a polaron peak, centered at 1020 nm (Figure 4a). The ground state bleach is evident below 670 nm. The 700–750 nm region shows little intensity at times longer than 100 ps, consistent with pristine μs-TAS data. It is worth noting that the pristine film’s polaron is evident on the earliest timescales (<100 fs) as a shoulder on the main exciton peak.

The PDTSiTTz:PC60BM 9:1 blend film shows the same singlet exciton and polaron peaks, but the relative amplitudes are very different (Figure 4c). The singlet exciton peak is very weak compared to the NIR polaron, and the 700 nm contribution is significantly more pronounced (consistent with the μs-TAS results and assigned to an additional polaron absorbance). Another observation is that, once the exciton has fully decayed at approximately 100 ps, the remaining polaron peak shows a red-shift over time of 21 nm (until a final time measurement of 7 ns; Figure S9). Such a red-shift is consistent with observation of the polaron peak at a longer wavelength of 1050 nm on microsecond timescales and may indicate the progressive localization of polymer polarons into more crystalline domains. The pristine polymer polaron shows no such red-shift, perhaps indicating that the polarons are already in a crystalline, isotropic, low energy environment.

The PDTSiTTz:ICTA 9:1 blend, in contrast, shows a spectral behavior intermediate between the pristine polymer and the PC60BM blend **(**Figure 4b). The exciton peak is significantly more pronounced than the 1020 nm polaron peak, as was observed in the pristine polymer: this is consistent with the lower efficiency of PL quenching. However, the 700 nm polaron contribution is much stronger for the ICTA blend than for the pristine blend. A smaller red-shift of the polaron band over time (11 nm) is observed for the ICTA blend. Since this red-shift is intermediate between the pristine and PC60BM blend, this may indicate that a high proportion of charges is created in crystalline, polymer-rich domains in this blend. Such a hypothesis is reasonable given the considerable ability of PDTSiTTz to generate charges in the absence of any acceptor and the lack of LUMO level offset with the acceptor that is present for the ICTA blend.

**Figure 5:**
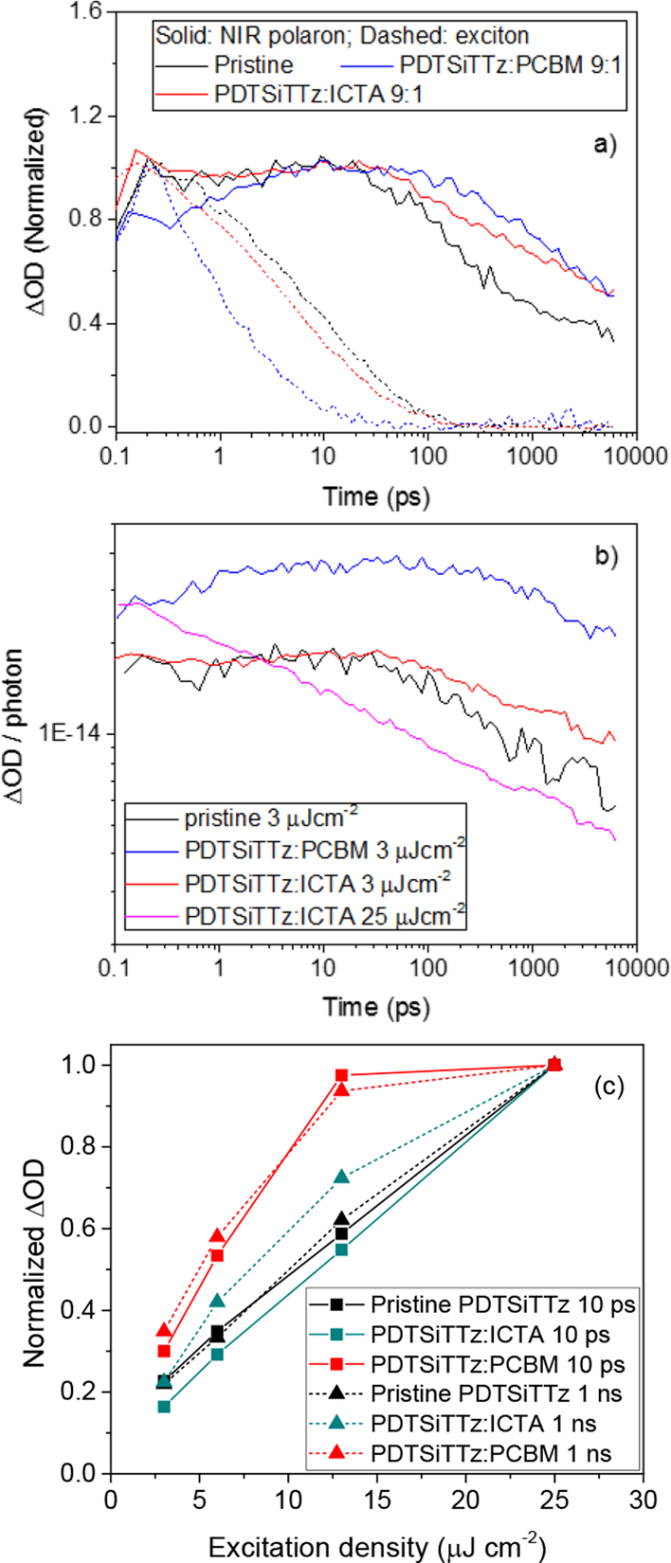
Femtosecond TA kinetics: (a) normalized kinetics comparing the pristine PDTSiTTz, PDTSiTTz:ICTA, and PDTSiTTz:PC60BM polymer exciton and polaron, using 6 μJ cm^–2^, and (b) kinetics normalized per photon absorbed for selected excitation densities. The excitation wavelength was 532 nm in all cases. The kinetics are derived from global analyses, where the exciton component absorbs maximally at 1270 nm and the polaron at 1020 nm. (c) Excitation density dependences of ΔOD at 10 ps and 1 ns for the NIR polaron spectral contribution.

A global analysis was performed for the three samples to extricate the individual signals for the NIR polymer polaron at 1020 nm and exciton at 1270 nm (Figure 4d–f). Even at the lowest excitation density of 3 μJ cm^–2^, the singlet exciton of the pristine PDTSiTTz exhibited a biexponential decay with lifetimes of 2.5 and 30 ps (Figure 4d and Figure S10). Although annihilation effects are possible (the invariance of the exciton kinetics between 3 and 6 μJ cm^–2^ makes this unlikely), the lack of monomolecular behavior is consistent with observation of polarons within the instrument time resolution (<150 fs) and thus suggests the presence of two exciton decay pathways. The shorter 2.5 ps lifetime is therefore assigned to charge separation of the exciton (consistent with the ultrafast appearance of the polaron peak in the pristine spectrum), while the longer lifetime is assigned to relaxation back to the ground state. Even so, an exciton lifetime of 30 ps is very short for such a wide band gap polymer. The exciton quenching induced by the addition of 10% PC60BM in the blend is evident in the substantially faster decay dynamics of the exciton (Figure 5a). In contrast, only a small decrease in exciton lifetime/s is observed for PDTSiTTz:ICTA. This suggests that the majority of charges generated in the ICTA blend are produced by pristine polymer domains, and the ICTA contribution as an electron acceptor is very small.

The inefficiency of ICTA as an electron acceptor for PDTSiTTz is also evident from a consideration of the ΔOD/photon amplitude at low excitation densities (Figure 5b), which shows the ICTA blend’s polaron to have the same magnitude as that of the pristine polymer. The two kinetic traces diverge after 40 ps, with the ICTA blend’s polaron showing clearly slower decay dynamics than the pristine polaron. As such, while the presence of the ICTA does not affect the charge photogeneration yield (and thus has a negligible contribution in its role as an electron acceptor), it has a large influence on the recombination dynamics. Indeed, it is apparent from 40 ps onward that PDTSiTTz:ICTA has slower polaron recombination kinetics than the PC60BM blend (Figure 5a)—a trend observed to continue into the microsecond timescale.

Excitation density-dependent analyses also reveal unusual PDTSiTTZ:ICTA behavior (Figure 4d−f). In the case of the pristine PDTSiTTz, the decay dynamics of the polaron are independent of excitation density until 7 ns, denoting geminate recombination (Figure 4d). For the PDTSiTTz:PC60BM polaron, an excitation density dependence is observed from approximately 10 ps, suggesting that this is the onset of bimolecular recombination in this blend (Figure 4f). The lack of geminate recombination is consistent with the high charge carrier densities at long times this polymer is capable of^2^ and the short CT state lifetime previously observed.^19^ Interestingly, while the PDTSiTTz exciton in the ICTA blend shows a very similar behavior to the pristine polymer exciton, the polaron shows a completely different kinetic behavior to either the pristine polymer or the PC60BM blend, with a significant excitation density dependence measured (Figure 4e). Such a strong dependence of decay kinetics on excitation density is not typically consistent with geminate recombination, despite the ICTA blend’s other similarities to the pristine polymer. The differences in decay kinetics for the PDTSiTTz:ICTA polaron are present prior to 10 ps, with the highest excitation density of 25 μJ cm^–2^ showing an immediate decay at the earliest times. Indeed, the 25 μJ cm^–2^ polaron kinetics can be fitted to a power law (Figure 5b). It is worth noting that the excitation density at which the ICTA begins to participate as an electron acceptor correlates with the excitation density at which the ultrafast recombination begins to be apparent.

To examine this excitation density dependence further, the ΔOD amplitude was measured at different times as a function of excitation density (Figure 5c). The PDTSiTTz:PC60BM polaron amplitude saturates with increasing excitation density irrespective of time, as expected for a second-order bimolecular process. The pristine PDTSiTTz polaron amplitude scales linearly with excitation density irrespective of time, indicative of a first-order process such as geminate recombination. In contrast, however, the PDTSiTTz:ICTA polaron amplitude shows a time-dependent behavior. At 10 ps, a linear increase is observed for PDTSiTTz:ICTA, but at 1 ns, this is replaced with a weak saturation. The implication of this is that the PDTSiTTz:ICTA blend shifts from first-order to second-order behavior as time progresses. The combination of a linearly increasing ΔOD and strongly changing kinetics with excitation density at early times observed for PDTSiTTz:ICTA is highly unusual.

## Discussion

**Figure 6:**
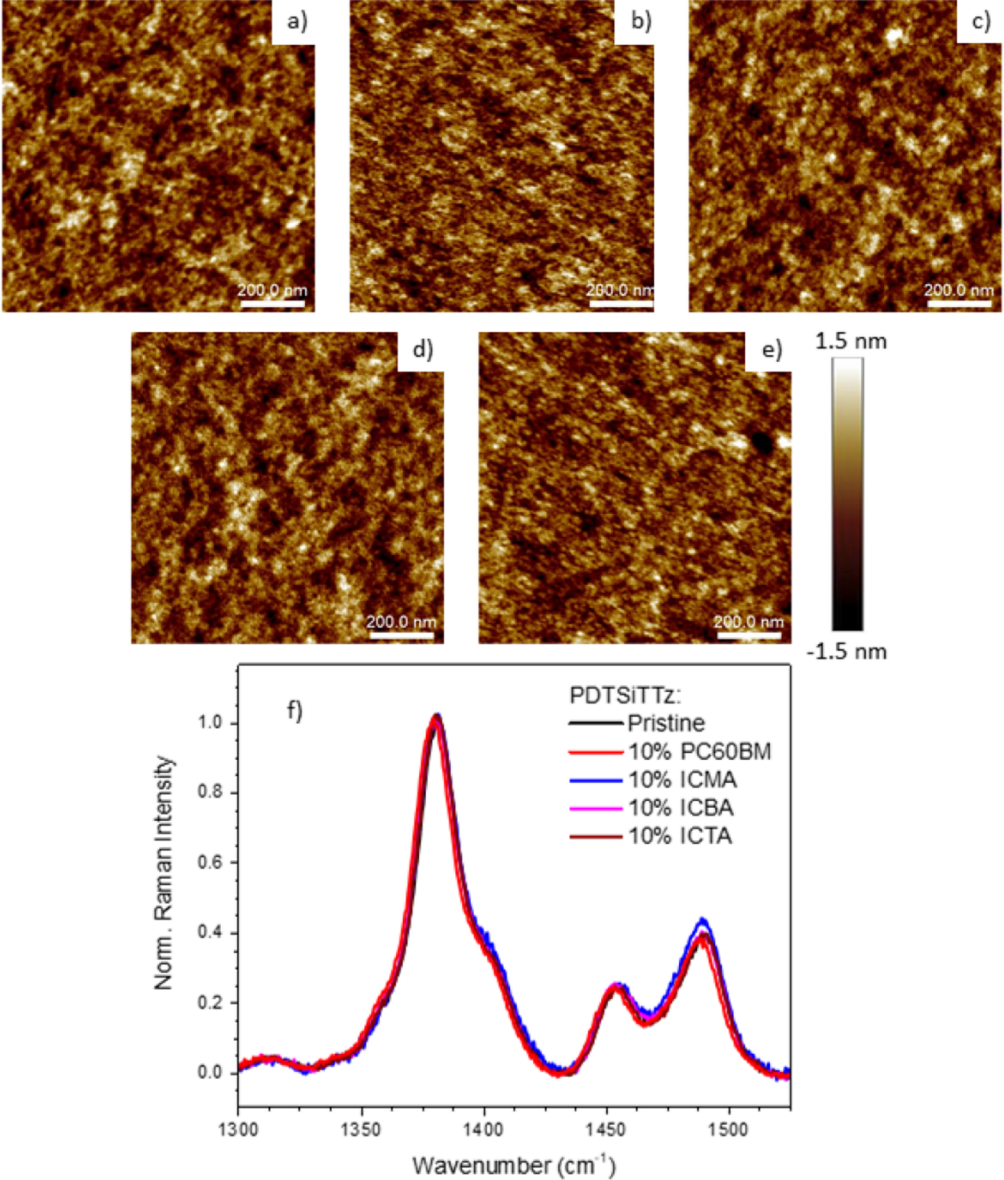
AFM images of (a) pristine PDTSiTTz, (b) PDTSiTTz:PC60BM, (c) PDTSiTTz:ICMA, (d) PDTSiTTz:ICBA, and (e) PDTSiTTz:ICTA films, where the blends all have 9:1 by weight ratios. The height scale bar for all samples in panels (a–e) is also shown. (f) Resonance Raman spectra, excited at 488 nm, of all 9:1 blends compared to pristine PDTSiTTz.

The TAS results show appreciable changes across the fullerene series for PDTSiTTz. The PDTSiTTz blends show progressively slower kinetics and a weaker thermalization as the fullerene is altered from PC60BM to ICTA on μs timescales. Furthermore, ps-TA data show PDTSiTTz:ICTA to have unusual excitation density-dependent recombination kinetics. To check if these changes could be related to bulk morphology, atomic force microscopy (AFM) on the film samples was performed (Figure 6a–e). The *R*_a_ values, the average of the absolute values of the surface height deviations measured from the mean plane, vary between 0.352 and 0.368 nm across the four blends, with PDTSiTTz:ICTA having the highest *R*_a_ of 0.368 nm. These blend *R*_a_ values are within ±0.013 nm of pristine PDTSiTTz (*R*_a_ = 0.355 nm). As such, no significant changes occurred relative to the pristine film and a low fullerene loading of 10% is largely preserving the morphology of the polymer. This was confirmed using Raman spectroscopy (Figure 6f), which is highly sensitive to structural and conformational changes. It is apparent that the Raman bands show negligible wavenumber shifts or intensity pattern changes when any of the fullerenes are added. It should be noted, however, that very subtle changes at the molecular level in terms of intermolecular orientations and/or separations are not likely to be observed by either technique.

**Figure 7:**
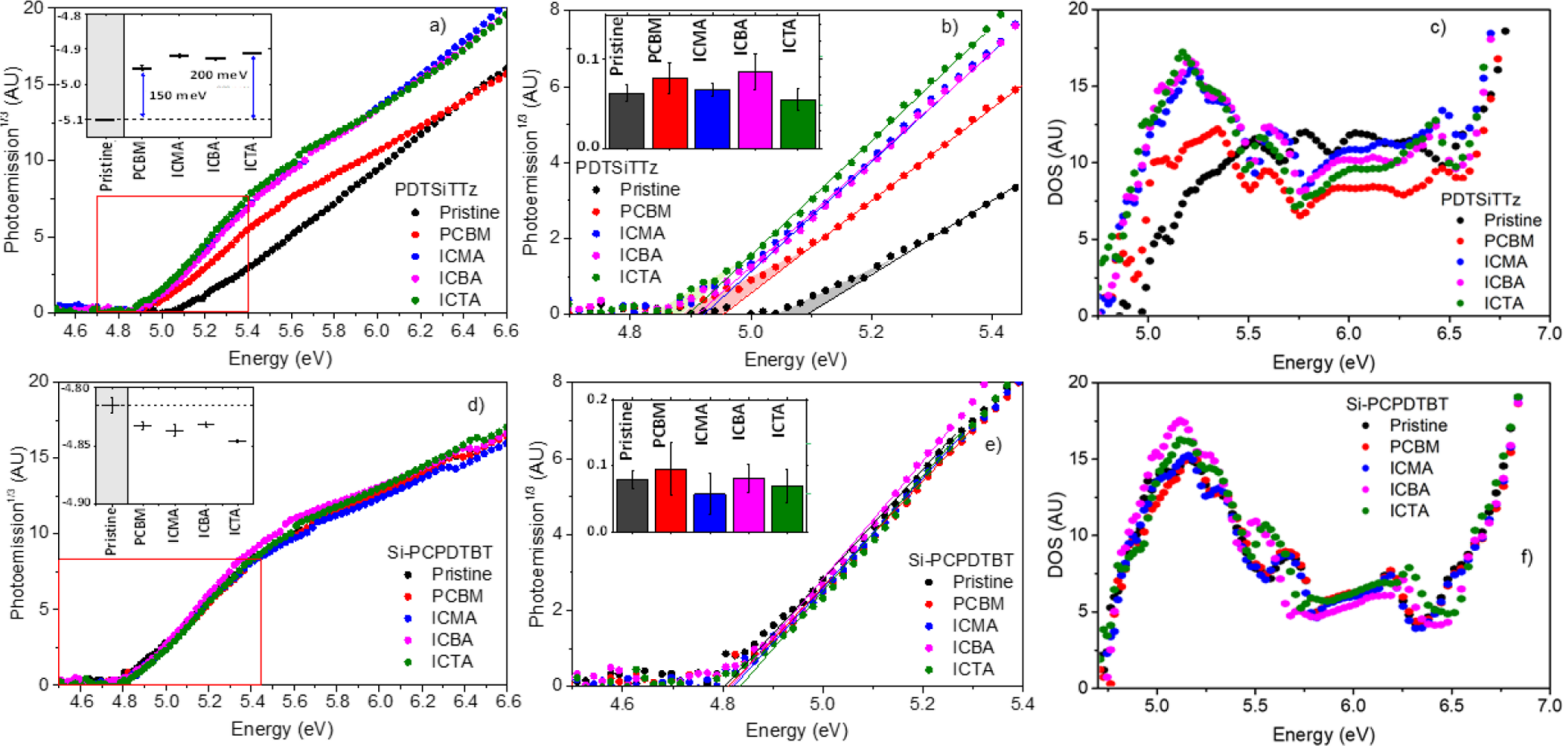
Ambient photoemission spectroscopy results for (a–c) PDTSiTTz and (d–f) Si-PCPDTBT and their 9:1 blends with PC60BM, ICMA, ICBA, and ICTA. Panels (a, d) show the raw data, panels (b, e) show an enlargement of the intercept region with the tail states highlighted, and panels (c, f) show the DOS derived from the first derivative of the APS signal. The insets in (a, d) show the corresponding HOMO values in eV for each system. The insets in (b, e) show the tail state density for each system.

Given the observation of fullerene-dependent polaron decay kinetics for PDTSiTTz, this may suggest an alteration of the density of states across the series. Power law kinetics in μs-TAS are consistent with models describing bimolecular recombination of dissociated charge carriers in the presence of an exponential distribution of localized states.^6^ Previous μs-TA studies have related the magnitude of the power law decay α component to the energetic depth of these localized states. To investigate this possibility, we employed ambient photoemission spectroscopy (APS), the results of which are shown in Figure 7. APS can determine HOMO energy levels, tail (trap) states, and density of states (DOS) of occupied molecular orbitals in neat polymers and their blend films. By linear fitting of APS photoemission signals, the HOMO level of the material can be obtained from the intercept. Tail states can be distinguished by integrating the area at energies below the linear fit. Furthermore, the distribution of the DOS is derived from the first derivative of the APS photoemission signal.

As shown in Figure 7d,e, the Si-PCPDTBT samples show relatively stable energetics upon blending. The HOMO level of neat Si-PCPDTBT is around −4.8 eV, and it deepens by only 0.03 eV in blends. This small change originates from the disorder introduced upon blending and is commonly seen in other organic blend systems. By converting APS data into DOS, we can see that the electronic state distribution is also stable upon blending (Figure 7f).

PDTSiTTz exhibits a HOMO level of −5.1 eV using APS (Figure 7a,b, slightly different from the CV results in Figure S1). A tail state density of 0.062 is within the typical range of polymers. However, when blended with 10% fullerene acceptors, an apparent shift of the polymer HOMO level is observed, with PDTSiTTz:ICTA showing the largest shift of 200 meV and a polymer HOMO of −4.9 eV. Intriguingly, there is no evidence of an increased density of tail states upon blending with the fullerenes. In contrast, however, the DOS itself shows significant changes. As evidenced from the DOS plots in Figure 7c, a large increase in DOS intensity at the PDTSiTTz band edge occurs upon blending with a fullerene, inducing the apparent HOMO shift. More specifically, the DOS shoulder at 5.2 eV in the pristine PDTSiTTz develops into a sharp peak at the same energy upon blending with the fullerene. Notably, the intensity enhancement of this feature is most prominent with the adducted fullerene (ICMA, ICBA, and ICTA) blends compared to PC60BM.

The pronounced changes in DOS for PDTSiTTz upon blending with fullerene are highly unusual. However, it was observed that the DOS values at the band edge of Si-PCPDTBT:ICTA and PDTSiTTz:ICTA are virtually identical in terms of amplitude, shape, and position (5.2 eV; Figure S11). The implication is that the HOMO (which is responsible for the DOS band edge) is primarily localized on the common dithienosilole donor unit of the polymer in both ICTA blend systems (and the pristine Si-PCPDTBT). This must not be the case in pristine PDTSiTTz, given that the pristine PDTSiTTz DOS is so different from PDTSiTTz:ICTA. Pristine PDTSiTTz may therefore have a more delocalized HOMO, either intramolecularly or intermolecularly, which could account for the broad and low-intensity DOS. An adjacent fullerene molecule may therefore act to localize the HOMO onto the dithienosilole unit of PDTSiTTz, potentially due to a change in the dielectric environment or by altering the interchain distance, thereby inhibiting HOMO delocalization onto adjacent chains. The latter possibility is supported by the fact that the localization is most prominent for ICBA and ICTA, the bulkiest of the fullerene series. Furthermore, B3LYP/def2-svp DFT calculations suggest that the HOMO levels of *in vacuo* PDTSiTTz and Si-PCPDTBT dimeric models are at approximately the same energy (−4.72 and −4.77 eV, respectively; Figure S12). Note that a change in interchain separation would not be expected to produce a change in the Raman spectra (Figure 6), provided that the same conjugation length is maintained.

**Figure 8:**
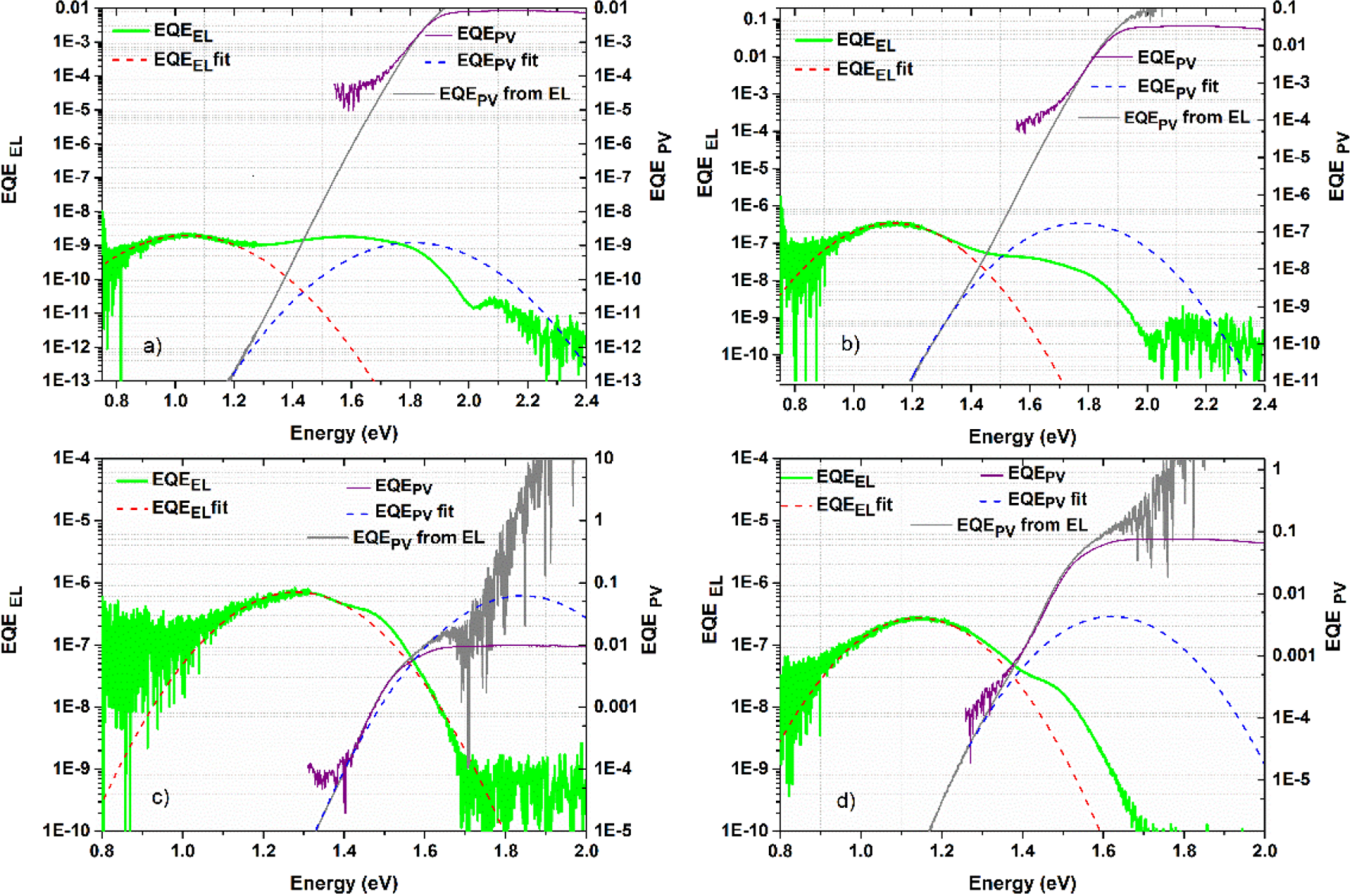
Electroluminescence and external quantum efficiency data for (a) pristine PDTSiTTz, (b) PDTSiTTz:PCBM (9:1), (c) pristine Si-PCPDTBT, and (d) Si-PCPDTBT:PCBM (9:1) films. The fits to each data set are shown, where the intersection of each inverted parabola provides the charge transfer state energy, *E*_CT_.

Expecting that these changes in the DOS band edge could have corresponding effects on CT state energies, we performed electroluminescence (EL) measurements on devices of the pristine polymers and their blends, exemplified by the PC60BM blends in Figure 8. Note that the device external quantum efficiency (EQE_PV_) values are very low because we are not examining device-optimized blend ratios. Instead, we are focusing on minimally doped films, in which charge generation and extraction will be sub-optimal, to perturb the polymer morphology as little as possible. Si-PCPDTBT and Si-PCPDTBT:PC60BM show standard behavior, with EL from the pristine polymer with a CT state energy, *E*_CT_, of 1.58 eV and EL from a lower energy intermolecular CT state in the PCBM blend with *E*_CT_ = 1.47 eV. In contrast, however, PDTSiTTz once again shows unusual behavior. Instead of simple EL arising from a single electronic state in the pristine polymer, there is clear evidence of two electroluminescent species in PDTSiTTz. One species, with its EL peak at approximately 1.85 eV corresponding to the optical band gap of PDTSiTTz, can be attributed to the S_1_ state. The other EL peak is at a much lower energy (∼1.05 eV), noting that the energy separation between the two EL peaks is inconsistent with vibronic structure. Such low energy—within the optical band gap—suggests an intragap CT state in the pristine material,^20^ possibly interchain in nature and/or at a grain boundary. The appearance of this low energy intragap state is consistent with the very short lifetime of the PDTSiTTz exciton and rapid appearance of charge carriers in the pristine polymer observed in the ps-TAS. This behavior is preserved upon blending with the fullerene, with both CT and S_1_ state EL peaks still present. However, rather than the expected decrease in *E*_CT_ upon blending (as seen for Si-PCPDTBT), an increase is observed for PDTSiTTz from *E*_CT_ = 1.40 to 1.51 eV. Intriguingly, it is the CT EL peak that appears to shift to higher energies for PDTSiTTz, likely because the weak interchain CT state in the pristine polymer is replaced by the stronger intermolecular donor/acceptor CT interaction. Indeed, it is also apparent that the PDTSiTTz and Si-PCPDTBT PC60BM blends have very similarly positioned EL peaks at ∼1.15 eV. This observation is consistent with the APS results suggesting that the fullerene acts to localize the PDTSiTTz HOMO, both shifting the HOMO to shallower energies and creating a similar HOMO (and thus CT state energy) to that observed for Si-PCPDTBT. This effect is not, however, observed in the UV–vis absorption spectra, likely because EL and APS techniques are much more sensitive to low energy states. Cyclic voltammetry data (Figure S1), however, do show an oxidation onset shift of 0.1 eV from the pristine PDTSiTTz to the blend. EL data for the other fullerenes are shown in Table S1.

Interestingly, these unusual EL and APS results for PDTSiTTz do not seem to be related to its non-Langevin characteristics since other non-Langevin systems such as the prototypical annealed P3HT:PCBM show neither pronounced double-peaked EL spectra^21,22^ nor HOMO shifts into the band gap upon blending.^23^ However, these aspects could still influence the recombination behavior of PDTSiTTz/fullerene blends. The PDTSiTTz:fullerene μs-TA data shows an apparent increase in trap depth through the fullerene series and incomplete thermalization of charges into these deep trap states for PDTSiTTz:ICBA and PDTSiTTz:ICTA. This increase in trap depth indicates an increasing contribution of the fullerene to the trapping characteristics. This could potentially relate to the increasingly large number of isomer possibilities from ICMA to ICTA. Previous computational works have shown that the higher number of isomer possibilities in ICTA may provide a larger distribution of energy levels, the lowest energy of which could produce the deep trap states.^24−26^ However, our APS data of the pristine fullerenes (Figure S13) clearly shows that, while the tail states of the fullerene DOS change little across the fullerene series, the breadth of the main DOS peak does increase, particularly from ICBA to ICTA. This suggests that the traditional viewpoint of “trapping” being dominated by tail states may not encompass the full picture and that the breadth of the main DOS peak may also have a strong influence. This hypothesis is also consistent with the PDTSiTTz APS results, which show a broadening of the PDTSiTTz DOS across the fullerene series (largely driven by the DOS intensity increase), which correlates with the decrease in recombination rate and enhanced trap depth (smaller α). Note that the driving force for recombination is likely to stay approximately the same as the shallower PDTSiTTz HOMO is compensated for by the shallower fullerene LUMO across the series.

The apparent localization of the HOMO (potentially onto a single PDTSiTTz chain segment) in the PDTSiTTz blends enables the increased contribution of the fullerene to the trapping characteristics of PDTSiTTz, noting that the decrease in the μs-TAS α across the PDTSiTTz series progressively approaches that of the Si-PCDTBT blends (α = 0.34). It has previously been postulated that ICTA forms very small clusters with an insulating indene shell,^27^ potentially reducing the likelihood of electron transfer. Such clusters could induce the observed PDTSiTTz HOMO localization, either via a change in the dielectric environment or by increasing the interchain distance. Furthermore, the slow charge carrier thermalization observed for the PDTSiTTz blends with ICBA and ICTA could be related to recombination involving the hole localized on the dithienosilole unit, rather than a lower energy hole delocalized over more of the polymer chain (as in pristine PDTSiTTz).

The trapping behavior shown by PDTSiTTz:ICTA is particularly interesting, given the apparent dominance of the fullerene. This is exacerbated by PDTSiTTz:ICTA being a zero offset system, with the ps-TAS data showing that charges are generated solely in the pristine polymer domains at low excitation densities. Only at high excitation densities does electron transfer to the ICTA become significant, and this is coupled with an ultrafast onset of charge carrier recombination. This ultrafast recombination may be related to both the large driving force for recombination from the ICTA LUMO and the polymer HOMO localization.^28^ Indeed, the linearly increasing ΔOD of PDTSiTTz:ICTA with excitation density (at early times; Figure 5c), coupled with strongly changing kinetics, is extremely unusual. One possibility is trap-assisted recombination^29−31^ (Shockley–Read–Hall recombination), which is known to be a first-order process that can occur on picosecond timescales. The ultrafast trapping in PDTSiTTz:ICTA at higher excitation densities leads to two phases of recombination: typical trap-limited bimolecular recombination on nanosecond timescales and trap-assisted recombination on picosecond timescales, both of which are influenced by the new intragap states induced by the fullerene interaction with the PDTSiTTz polymer chain. In contrast, the greater delocalization and driving force for charge separation in PDTSiTTz:PC60BM enable both slower recombination and greater charge carrier densities.

## Conclusions

Using μs-TAS, we have shown that PDTSiTTz shows diverse bimolecular recombination kinetics across the fullerene series of PC60BM, ICMA, ICDA, and ICTA. Notably, the μs-TA kinetics of the polymer polaron becomes progressively slower across the series, with reduced charge carrier thermalization in the ICBA and ICTA blends. In contrast, Si-PCPDTBT blends show invariant polymer polaron μs decay kinetics. Furthermore, ps-TAS on marginal energy offset blend PDTSiTTz:ICTA shows evidence of an ultrafast onset of trap-assisted recombination at high excitation energies that is correlated with the ICTA beginning to participate as an electron acceptor. These results have been explained using a combination of device electroluminescence and ambient photoemission spectroscopy. The electroluminescence measurements showed an unusual double peak in pristine PDTSiTTz, attributed to a low energy intragap charge transfer state in addition to the standard S_1_ state. Given the lack of acceptor present, this intragap CT state is likely to be interchain in nature, possibly at a grain boundary. The APS results also showed unusual results for PDTSiTTz, with the DOS changing considerably from the pristine polymer to the blends. While the pristine PDTSiTTz showed a broad, low-intensity DOS, the fullerene blends showed an intensity enhancement of a low energy feature at 5.2 eV, culminating in the PDTSiTTz ICBA and ICTA blends presenting a virtually identical DOS to Si-PCPDTBT and its blends. We have attributed this to a localization of the PDTSiTTz HOMO from its delocalized, interchain nature in the pristine material to a dithienosilole-focused HOMO in the blends, likely a result of the bulky fullerene increasing interchain separation. Intriguingly, the increasing intensity of the DOS across the PDTSiTTz blends series led to an apparent progressive shift of the polymer HOMO to shallower energies, but the density of tail states remained the same. As such, this broadening of the PDTSiTTz DOS across the fullerene series correlates with the observed decrease in recombination rate and increased trap depth. This suggests that the traditional viewpoint of trapping being dominated by tail states may not encompass the full picture and that the breadth of the main DOS peak may also have a strong influence.

## Supporting Information Available

Additional experimental data, including cyclic voltammetry, steady state absorbance and fluorescence, TAS oxygen dependence, solution TAS data, full TAS assignment description, μs-TAS excitation density dependence, computational results, pristine fullerene APS data, and CT energies extracted from EL data (PDF)
