# Supplementary material for: Insight into the Origin of Trapping in Polymer/Fullerene Blends with a Systematic Alteration of the Fullerene to Higher Adducts
Source: J Phys Chem C Nanomater Interfaces. 2022 Jan 31;126(5):2708–19. doi: 10.1021/acs.jpcc.1c10378 (PMC9097530; doi:10.1021/acs.jpcc.1c10378)
Supplement: Supplementary file 1 — jp1c10378_si_001.pdf [file jp1c10378_si_001.pdf]

Supporting Information

Insight into the Origin of Trapping in Polymer / Fullerene Blends with a Systematic Alteration of the Fullerene to Higher Adducts

Jose Marin-Beloqui,[a,§] Guanran Zhang,[b,#] Junjun Guo,[a] Jordan Shaikh,[a] Thibaut Wohrer,[a,c] Seyed Mehrdad Hosseini,[d] Bowen Sun,[d] James Shipp,[e] Alexander J. Auty,[e] Dimitri Chekulaev,[e] Jun Ye,[c] Yi-Chun Chin,[f] Michael Sullivan,[c] Attila J. Mozer,[b] Ji-Seon Kim,[f] Safa Shoaee,[d] and Tracey M. Clarke.[a]*

[a] Department of Chemistry, University College London, Christopher Ingold Building, London WC1H 0AJ, United Kingdom.

[b] ARC Centre of Excellence for Electromaterials Science, Intelligent Polymer Research Institute, University of Wollongong, North Wollongong, NSW 2500, Australia

[c] Institute of High Performance Computing A*STAR, Singapore 138632, Singapore

[d] Optoelectronics of Disordered Semiconductors, Institute of Physics and Astronomy, University of Potsdam, Karl-Liebknecht-Strasse 24-25, 14476 Potsdam-Golm, Germany

[e] Department of Chemistry, The University of Sheffield, Sheffield, S3 7HF, United Kingdom.

[f] Department of Physics and Centre for Processable Electronics, Imperial College London, London, SW7 2AZ, United Kingdom.

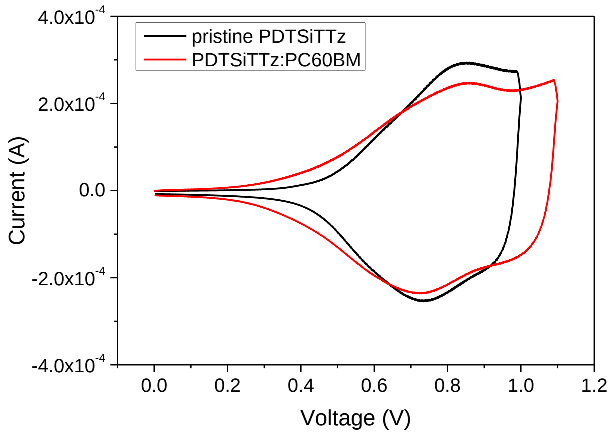

Figure S1. Cyclic voltammograms of pristine PDTSiTTz and its 1:2 blend with PC60BM. All were measured as solid thin films on ITO substrates in a deoxygenated acetonitrile/TBAP medium, using a $Ag/AgNO_3$ reference electrode and a scan rate of 100 mV s^{-1}.

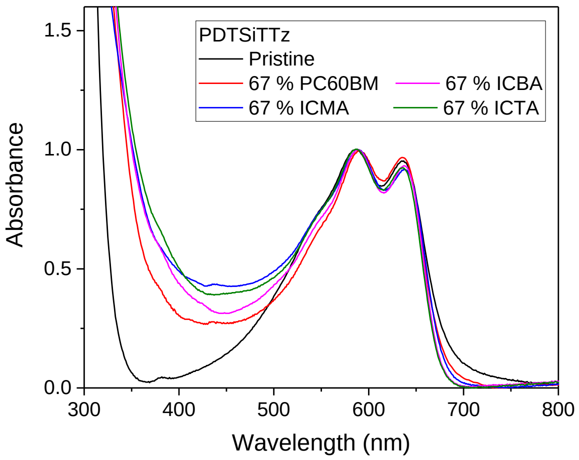

Figure S2. Steady state absorption spectra of thin films of PDTSiTTz and its 1:2 blends with the fullerenes PC60BM, ICMA, ICBA, and ICTA.

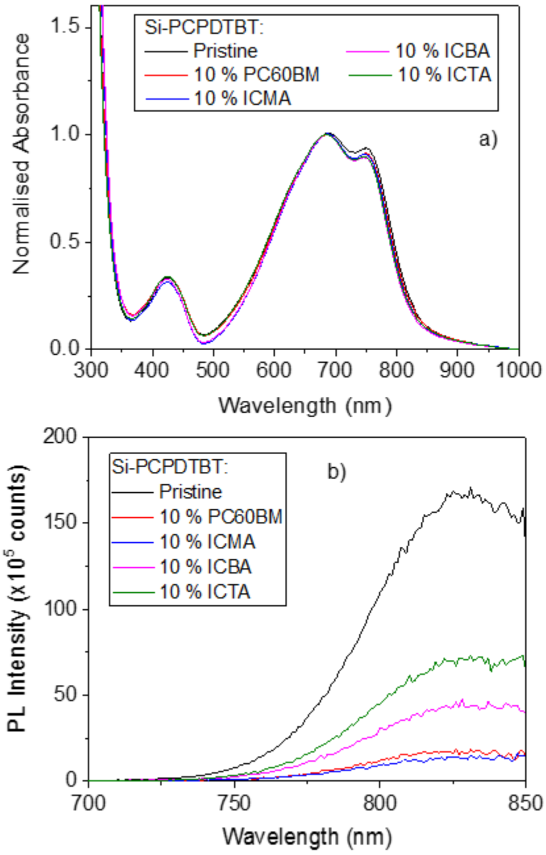

Figure S3. Steady state (a) absorption and (b) photoluminescence spectra of thin films of Si-PCPDTBT and its 9:1 blends with the fullerenes PC60BM, ICMA, ICBA, and ICTA.

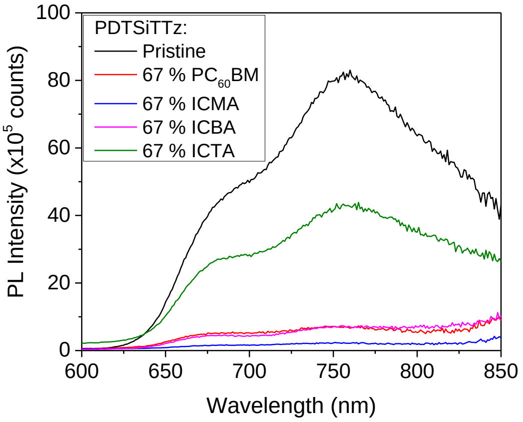

Figure S4. Steady state photoluminescence spectra of thin films of PDTSiTTz and its 1:2 blends with the fullerenes PC60BM, ICMA, ICBA, and ICTA.

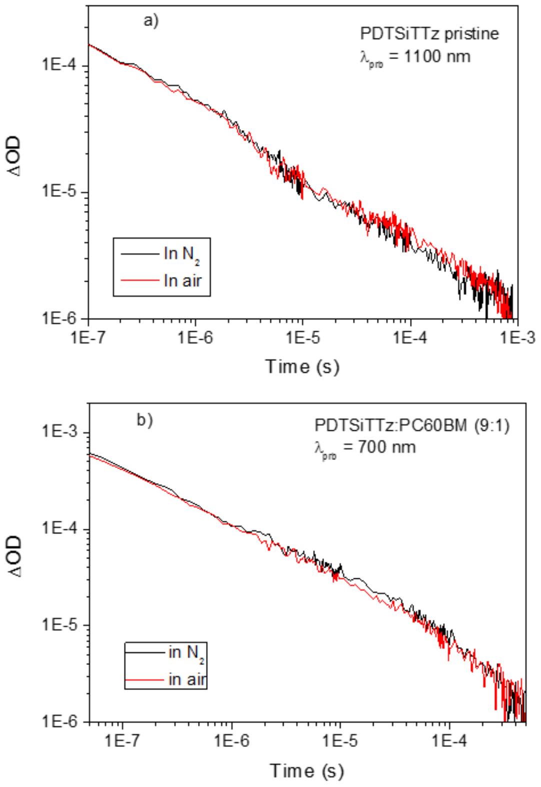

Figure S5. Microsecond TAS kinetics of (a) pristine PDTSiTTz film, probed at 1100 nm, and (b) PDTSiTTz:PC60BM (9:1) at 700 nm, both showing a lack of oxygen dependence. Excitation wavelength is 532 nm, pump excitation density 10 μJ cm^{-2}.

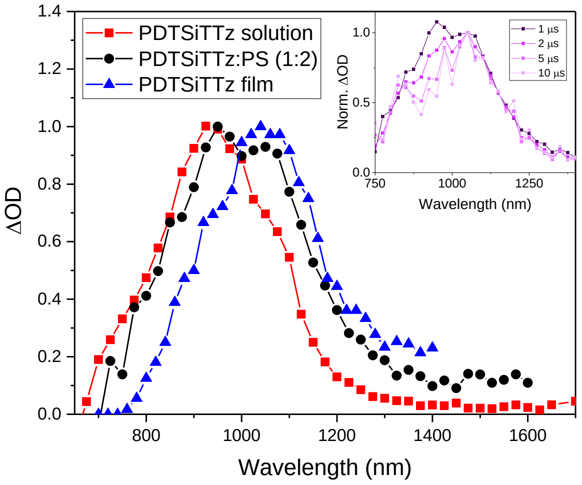

Figure S6. Normalised μs-TA spectra of PDTSiTTz in solution, film, and blended with inert polystyrene (PS). The inset shows the PDTSiTTz:PS spectral evolution, with evidence of two distinct species with different kinetics present. Excitation wavelength 532 nm, pump excitation density 10 μJ cm^{-2}.

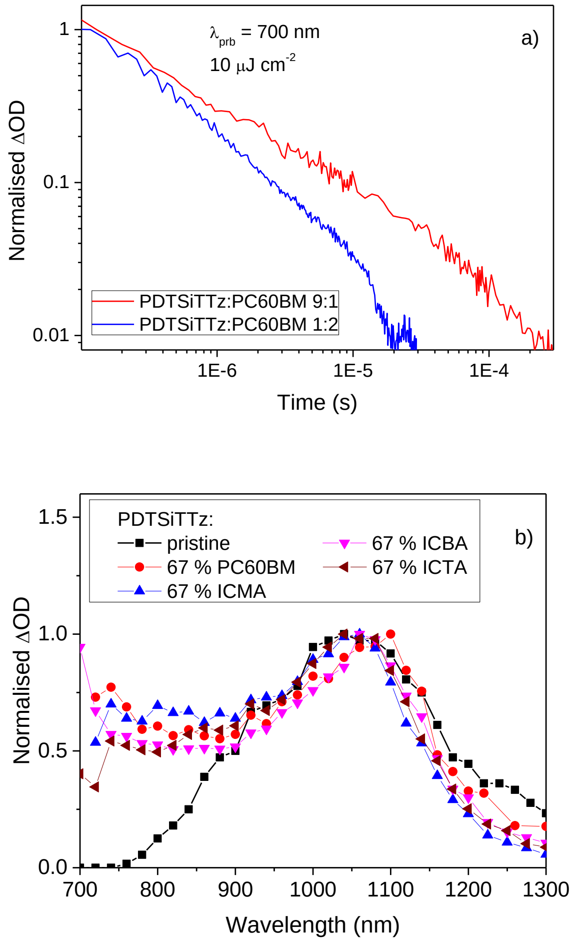

Figure S7. (a) Normalised μs-TA kinetics for PDTSiTTz blended in 9:1 and 1:2 weight ratios, probed at 700 nm. (b) Normalised μs-TA spectra of thin films of PDTSiTTz and its 1:2 blends with the fullerenes PC60BM, ICMA, ICBA, and ICTA. Excitation wavelength 532 nm, pump excitation density 10 μJ cm^{-2}.

Addition of any of the fullerenes to create the PDTSiTTz:fullerene 9:1 blend films also results in the 1050 nm transient absorption. The unshifted 1050 nm band in the blend films is therefore also assigned to the PDTSiTTz polaron, as reported previously.[28] Unlike pristine PDTSiTTz, however, there is also a tail of absorption below 750 nm in all PDTSiTTz blends.

One possibility is that this additional absorption feature below 750 nm is related to the fullerene: the fullerene anion or triplet state. In the case of PC60BM, the anion is located at 1020 nm while the triplet absorbs at 720 nm (another species suggested in the literature, the PC60BM cation, absorbs at ~ 900 nm[29]). Given the 700 – 750 nm range where this additional absorbance is observed in the PDTSiTTz:fullerene 9:1 blends, the fullerene triplet is an obvious possibility. However, the decay dynamics probed at 700 nm still follow power law behaviour and no air sensitivity (oxygen quenching) is observed (**Figure S5**). Both these observations contradict a fullerene triplet assignment. Furthermore, the transient bands of fullerenes are known to have very weak extinction coefficients compared to that of the conjugated polymers. Given that this 700 – 750 nm absorption tail is of a comparable amplitude to the main polymer polaron peak, this is another indicator that it cannot be attributed to the fullerene triplet state. However, the magnitude of this absorption relative to the

polymer polaron peak increases when the concentration of fullerene increases to 67 % (**Figure S7**). Although not the fullerene triplet, this absorption band is clearly influenced by the fullerene in some way.

The absorption tail below 750 nm observed for the 9:1 PDTSiTTz blends has also previously been observed in faster time scale TAS experiments of 1:2 PDTSiTTz:PCBM blends as a band at 750 nm that decays faster than the 1050 nm band.[28] Both bands show power law decay dynamics, suggesting bimolecular recombination of charge carriers. Such bimodal polaron band behaviour has been observed in other polymer/PCBM blends and has typically been assigned to delocalised and localised polarons on the basis that the localised (trapped) polarons should recombine more slowly than delocalised polarons.[13] For P3HT:PCBM, an additional polaron absorption at 800 nm has also been observed on sub-nanosecond timescales, which also increases in amplitude with fullerene concentration and was assigned to monomolecular recombination of localised P3HT polarons bound to fullerene anions in disordered amorphous domains.[30] Furthermore, a low bandgap, ambipolar polymer, XIND, displayed bimodal polaron behaviour in fullerene or P3HT blends, with both bulk and interfacial polarons being clearly observed. In this case, the bulk XIND polarons at 1400 nm exhibited rapid decay dynamics while the interfacial XIND polarons decayed much more slowly. It is clear that bimodal polaron behaviour is more complex than previously conceived, and that the origin is dependent on the material system and nanomorphology present.

In the case of PDTSiTTz, both the relative amplitude and decay rate of the PDTSiTTZ blends' 750 nm band increase at the larger fullerene concentration of 1:2, which is consistent with charge recombination occurring in an amorphous mixed phase including both polymer and fullerene domains. Recombination in this case would become faster and more prominent as the availability of recombination sites (polymer/fullerene interfaces) increases. Conversely, the 1050 nm band, which is also present for the pristine polymer, is likely to involve bimolecular recombination occurring in predominantly pure (semi-crystalline) polymer regions. The previous assignment of "trapped" polarons undergoing slow bimolecular recombination for this 1050 nm band is likely to still be valid. It has been proposed by several groups that crystalline regions of polymer can in fact act as trap sites[31-33] since they have a lower ionisation potential and thus can act as energy sinks for holes, requiring thermal activation up the energy gradient to reach a polymer/fullerene interface for recombination. This may in fact have a contribution to the "trapping" implicit in the power law kinetics of bimolecular recombination. Note that these 1050 nm polarons localised in crystalline regions of the PDTSiTTz film decay much slower than that observed for the "bulk" polarons of XIND previously mentioned. This is likely due to the fact the fast XIND kinetics have a component of geminate recombination arising from the ambipolar nature of that polymer.

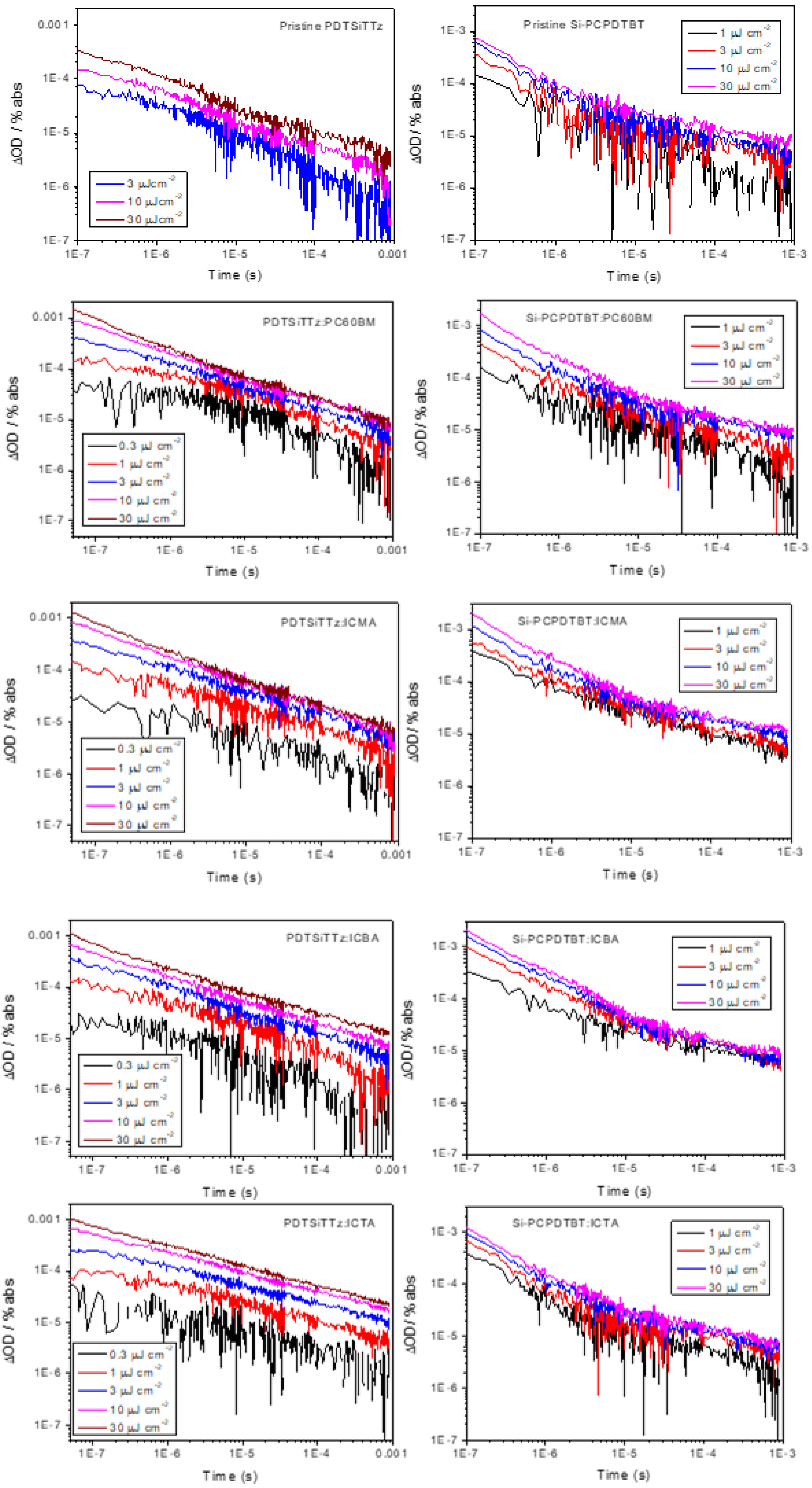

Figure S8. Excitation density dependent μs-TA kinetics for PDTSiTTz (left column) and Si-PCPDTBT (right column) pristine thin films and their blends with the fullerenes PC60BM, ICMA, ICBA, and ICTA. Excitation wavelength 532 nm, pump excitation density 10 μJ cm^{-2}, probe wavelength of 1100 nm for

PDTSiTTz. Excitation wavelength 600 nm, pump excitation density 10 μJ cm^{-2}, probe wavelength of 1300 nm for PDTSiTTz.

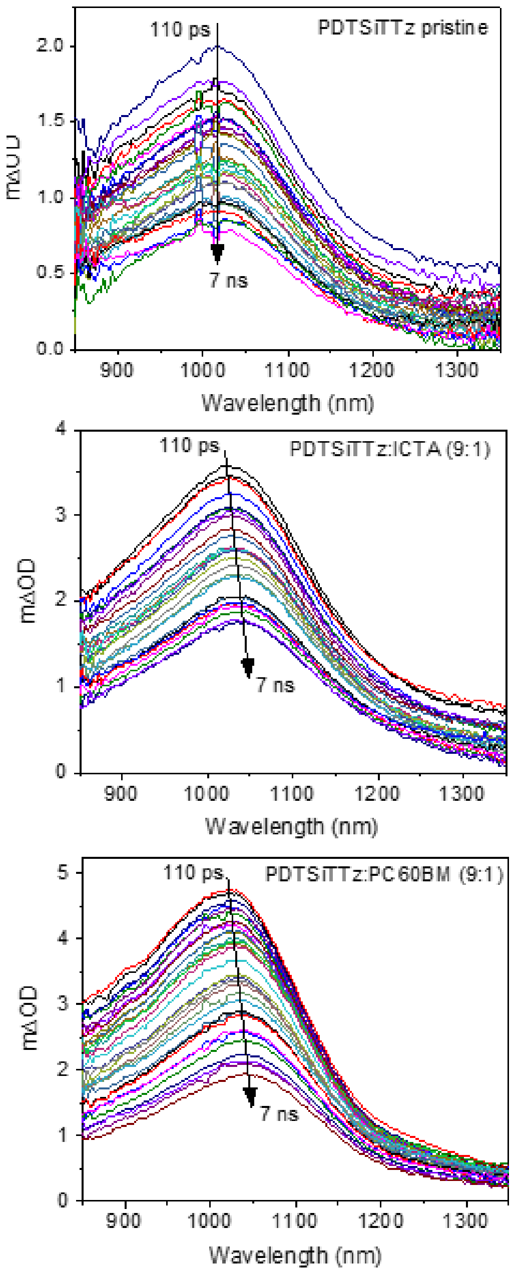

Figure S9. Picosecond TA spectra of the polymer polaron for pristine PDTSiTTz, PDTSiTTz:ICTA, and PDTSiTTz:PC60BM (9:1). Excitation wavelength 532 nm, pump excitation density 25 μJ cm^{-2}.

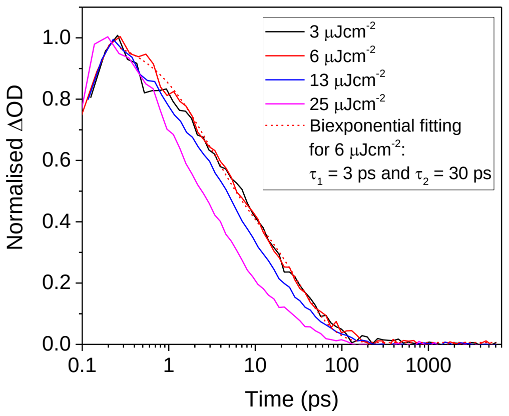

Figure S10. PDTSiTTz singlet exciton decay dynamics extracted from global analysis of the ps-TAS data as a function of excitation density. A biexponential fit to the data is also shown.

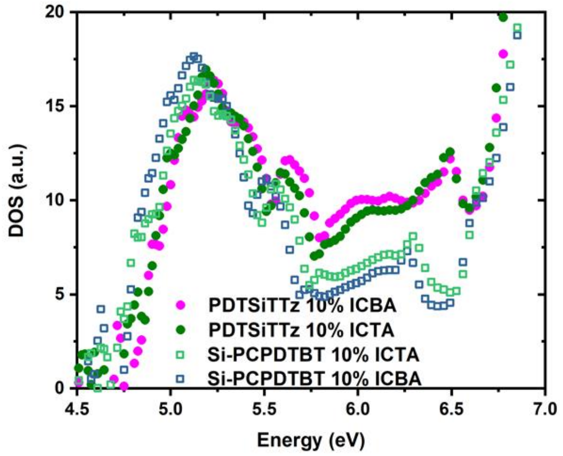

Figure S11. DOS extracted from APS data of the blends of PDTSiTTz and Si-PCPDTBT with ICBA and ICTA (9:1), showing their similarity.

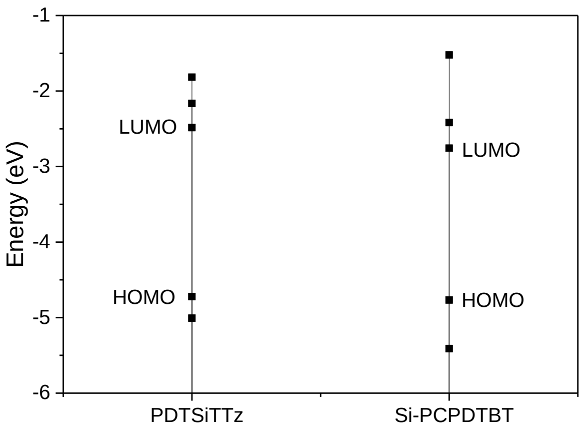

Figure S12. DFT calculations (B3LYP/def2-svp) of the MO energy levels for PDTSiTTz and Si-PCPDTBT, using a dimer model for each polymer.

Table S1. CT state energies and reorganisation energies (λ) obtained from electroluminescence data for PDTSiTTz and Si-PCPDTBT and their fullerene blends.

	**CT energy (eV)**	**λ (eV)**
neat PDTSiTTz	1.40 ± 0.05	0.38 ± 0.05
PDTSiTTz:PCBM	1.51 ± 0.05	0.40 ± 0.05
PDTSiTTz:ICMA	1.47 ± 0.04	0.32 ± 0.05
PDTSiTTz:ICBA	1.60 ± 0.03	0.38 ± 0.08
PDTSiTTz:ICTA	1.55 ± 0.03	0.38 ± 0.08
neat Si-PCPDTBT	1.58 ± 0.03	0.31 ± 0.03
Si-PCPDTBT:PCBM	1.47 ± 0.04	0.34 ± 0.04
Si-PCPDTBT:ICMA	1.33 (1.45) ± 0.03	0.25 (0.30) ± 0.02
Si-PCPDTBT:ICBA	1.49 ± 0.02	0.24 ± 0.02
Si-PCPDTBT:ICTA	1.50 ± 0.05	0.20 ± 0.04

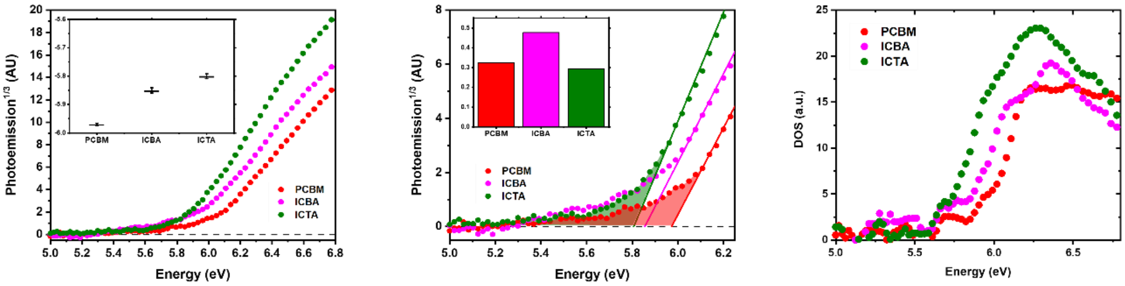

Figure S13. APS data of films of the pristine fullerenes PC60BM, ICBA, and ICTA. Note that films of ICMA were of very poor quality and thus APS data could not be measured.
